# The Rabbit as a Model for Studying Lung Disease and Stem Cell Therapy

**DOI:** 10.1155/2013/691830

**Published:** 2013-04-08

**Authors:** Nurfatin Asyikhin Kamaruzaman, Egi Kardia, Nurulain ‘Atikah Kamaldin, Ahmad Zaeri Latahir, Badrul Hisham Yahaya

**Affiliations:** Cluster for Regenerative Medicine, Advanced Medical & Dental Institute (AMDI), Universiti Sains Malaysia, Bandar Putra Bertam, 13200 Kepala Batas, Malaysia

## Abstract

No single animal model can reproduce all of the human features of both acute and chronic lung diseases. However, the rabbit is a reliable model and clinically relevant facsimile of human disease. The similarities between rabbits and humans in terms of airway anatomy and responses to inflammatory mediators highlight the value of this species in the investigation of lung disease pathophysiology and in the development of therapeutic agents. The inflammatory responses shown by the rabbit model, especially in the case of asthma, are comparable with those that occur in humans. The allergic rabbit model has been used extensively in drug screening tests, and this model and humans appear to be sensitive to similar drugs. In addition, recent studies have shown that the rabbit serves as a good platform for cell delivery for the purpose of stem-cell-based therapy.

## 1. Introduction

Public awareness of lung disease has grown tremendously in the last several decades. People can experience either acute or chronic disease. Acute lung disease describes conditions of abnormal lung function when exposed to certain stimuli, and it can have severe effects on human health. Acute lung injury (ALI) and acute respiratory distress syndrome (ARDS) are clinical syndromes that are defined by varying degrees of ventilation perfusion mismatch, acute hypoxemic respiratory failure, bilateral pulmonary infiltrates (oedema and normal cardiac filling pressure), and poor lung compliance [1, 2]. This failure can be classified as life-threatening respiratory failure due to lung injury caused by a variety of precipitants.

**Figure 1:**
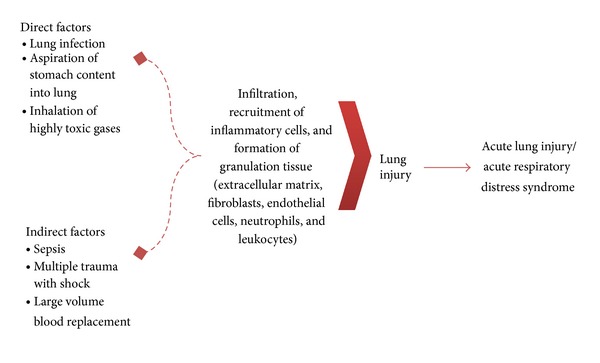
Direct and indirect factors that contribute to the onset of acute lung disease, especially acute lung injury and acute respiratory distress syndrome.

The development of ALI/ARDS can be either direct or indirect, with development patterns differing in terms of the route of the injury to the lung (Figure 1). Pulmonary oedema refers to the influx of protein-rich fluid into the alveolar spaces resulting from the increased permeability of the alveolar-capillary barrier [3], and it is often observed in the early stage of ALI/ARDS. Oedema is categorised as a direct factor when the injurious agent reaches the lung through the airways or by trauma to the chest and as an indirect factor when the injurious agent reaches the lung through the bloodstream [4].

In the early stage of ALI/ARDS development, the pathology of the patient's lung is accompanied by increased capillary permeability, alveolar and pulmonary oedema, and necrosis of the alveoli [4, 5]. Common clinical features in patients with pulmonary oedema are shortness of breath, coughing up blood or bloody froth, difficulty breathing when lying down, a feeling of “air hunger” or drowning, and grunting or wheezing sounds with breathing.

ALI, which is the mildest form of ARDS, develops in the early stages of lung disease. Any stimulus that can initiate systemic or local inflammation can trigger the onset of ALI in a person. ARDS is characterised by the diffusion of alveolar damage, alveolar capillary leakage, and protein-rich pulmonary oedema, all of which lead to clinical observations of poor lung function, severe hypoxemia, and bilateral infiltrates on chest radiographs [2]. ARDS often develops in patients diagnosed with sepsis, pneumonia, and trauma that has been treated with multiple transfusions. Patients diagnosed with ARDS are usually mechanically ventilated when the attacks occur. Common clinical conditions associated with the onset of ARDS are pneumonia, aspiration of gastric contents, pulmonary contusion, fat emboli, near drowning, inhalation injury, and reperfusion pulmonary oedema after transplantation or pulmonary embolectomy [5]. Workers in chemical producing factories can also develop ALI/ARDS due to inhalation of noxious fumes. 

Other than harsh chemical agents and biological factor, physical force on airway lumen also can induce hyperresponsiveness. In a study done by Latahir and Yahaya [6], they used a novel brushing technique via tracheal perturbation using an endotracheal tube to induce injury on the upper airway. This technique applied amount of forces on the wall of the trachea, which caused bleeding in the area of injury. Later on, during the recovery period, the recruitment of neutrophils into the area of injury will lead to the onset of acute lung injury. This novel technique can be used as an alternative method to induce acute lung injury on airway lumen without doing minor surgery which requires skill.

Asthma, chronic obstructive lung disease (COPD), chronic bronchitis, emphysema, bronchiectasis, and cystic fibrosis are the primary examples of the pathological consequences of chronic lung disease. Smoke inhalation is the main aetiological cause, but occupational environment and underlying genetic makeup also play significant roles in developing chronic lung disease. These diseases are usually associated with development of airway inflammation, airway hyperresponsiveness (AHR), and mucus overproduction. COPD refers to conditions characterised by chronic or recurrent obstruction of air flow, including chronic bronchitis and emphysema. Chronic bronchitis results from recurrent episodes of acute bronchitis or persistent and noninfective irritation of bronchial mucosa. This disease is usually associated with emphysema, a condition characterised by persistent dilatation of air spaces and destruction of their walls [7]. Asthma is characterised by a variety of features, including reversible airway obstruction, airway inflammation, and increased airway responsiveness to both physical and chemical stimuli [8].

## 2. Rationale for Using Animal Models to Study Lung Disease

Lung diseases are extremely prevalent worldwide, thus the development of prevention strategies and new treatment methods to reduce the worldwide burden and increase personal quality of life is crucial. As for any other disease, clinical investigation and epidemiological studies are needed to advance knowledge and improve disease management [9]. A biological model is needed to study the mechanisms that underlie the development of lung disease at both cellular and molecular levels.

Human models of ALI have been reported in several case studies. Human models were used in a clinical trial to develop and test a novel therapeutic agent targeted to cure ALI/ARDS [10]. However, there are drawbacks to use humans as research subjects. Humans can suffer from other disease during the experimental period, and there are risks associated with experimental procedures (e.g., inducing a low level of inflammatory response). Therefore, healthy volunteers must be thoroughly educated about the potential risk of participating in a given study. Various ethical and pathological issues also limit the use of humans as an *in vivo* model.

Thus, animals are preferable as an experimental model for modelling the human respiratory system. They provide an experimental setting that allows researchers to study the interaction between the immune system and a functioning respiratory system. Choosing an ideal animal model for use in lung disease studies must follow several criteria. The most important is the ability of the model to reproduce the principal aspect of human lung disease. The model also should be able to imitate the sensitised human lung conditions in terms of the nature of the injury and any disease-induced changes that occur at biological, physiological, and pathological levels. 

For example, when modeling ALI/ARDS in an animal model, the model should be able to mimic the sensitised human lung condition, which includes injury at the alveolar-capillary membrane, neutrophil-induced inflammation, and increased permeability of pulmonary oedema (Table 1). To simulate human ALI/ARDS, the animal model also should reproduce the acute lung injury to the epithelial and endothelial barriers in the lungs and the acute inflammatory response in the air spaces [2]. 

For chronic lung diseases such as asthma, an effective model should reproduce the principal aspects of human asthma, which include immunoglobulin E (IgE)-mediated sensitivity to antigens, acute bronchoconstriction, increased airway resistance, chronic inflammation of the airways, Th2 cytokine production, eosinophilia, late phase airway obstruction, enhanced mucus secretion, decreased mucociliary clearance, airway wall remodelling, and smooth muscle hyperplasia [11]. In addition, an ideal animal model must have the ability to develop disease and evolve injury over a prolonged period of time [12].

In order to mimic the pathophysiology of a human disease, a large-sized animal is more suitable than a small-sized animal because the complexity of its organ structure is more similar to that of humans. Moreover, observation of disease development is much easier in larger animals. Different animal models have been used to study lung disease, including mice, guinea pigs, and rabbits. Each animal possesses certain advantages and disadvantages as a model of lung disease. Below are descriptions of the pros and cons of various animals for use in studying lung disease, particularly asthma (Table 2).

### 2.1. Mouse

The mouse is an ideal model for studying most diseases because we have a detailed understanding of mouse genetics [20, 21]. The mouse has become the most popular animal for modelling allergic airway responses such as asthma. IgE is the primary allergic antibody in mice, which is similar to the situation in humans; therefore, this species is thought to be suitable for investigation of humoral immune factors in the development of allergic airway disease [8, 9]. Moreover, numerous immunological reagents and tools for use in mouse studies are available, and they offer the opportunity to explore detailed mechanisms of allergic reactions [8, 9, 22]. In addition, the availability of knock out or transgenic species makes mice a preferred model for any study related to gene manipulation [8]. Furthermore, mice are relatively cheap and easy to breed and have a short gestational period, thus allowing large studies to be conducted [8, 22].

Despite these advantages, mice and humans have considerable physiological differences. The most relevant differences for lung disease studies are differences in lung anatomy and airway musculature. In the mouse, vasculature is the primary target of anaphylactic response, whereas the lung is the primary target for anaphylactic response in humans [9]. In addition, the poorly developed airway musculature in mice suggests that developing a physiological model of pulmonary responsiveness is inappropriate in this species [9].

Plasma exudation is a cardinal sign of bronchial asthma and allergic rhinitis [23]. In humans, numerous plasma-derived, inflammatory-, repair-, leukocyte-, and growth factor-active proteins, irrespective of size, are distributed throughout the airway tissues. In contrast, Gelfand [23] reported that mice showed little plasma exudation, especially in the late phase response even after allergy loading. In sensitised mice, the inflammatory response to antigen challenge usually results in a massive influx of inflammatory compounds, dominated by eosinophils into the airways [22]. From an immunological point of view, eosinophils rarely degranulate in mouse models of asthma, whereas they readily degranulate in humans. Because both plasma exudation and eosinophil degranulation may correlate with disease severity, it appears that asthma-like symptoms are more or less lacking in the allergic mouse model [23]. In addition, the mast cells of mice and rodents generally release serotonin, but this is not thought to play a role in human asthma. For these reasons, the mouse model is not suitable for studying asthmatic disease. Allergic mice also do not exhibit spontaneous AHR, and smooth muscle hyperplasia is not easily demonstrated [8]. 

### 2.2. Guinea Pig

Because guinea pigs are small and docile animals, for over 100 years, they have been the most widely used test systems for contact hypersensitivity to chemical irritants and proteins. Some researchers may assume guinea pigs as an ideal model to study asthmatic disease because they develop well-characterised early and late phase airway responses to allergen challenge following sensitisation. This allows mechanistic investigation of each reaction as well as the relationship between the two responses [9, 20]. The associated pulmonary inflammatory responses, which involve both eosinophils and neutrophils, are consistent with asthma [8]. 

This species produces both IgG1 and IgE antibodies, with IgG1 being more dominant than IgE. From an immunological point of view, this presents difficulties in studies of mechanisms of humoral responses to allergens [9]. In addition, mechanistic studies, especially studies involving genetics, using the guinea pig model are limited due to the low number of inbred strains available. A limited number of species-specific reagents is available, thus making it difficult to identify and isolate particular cell types such as lymphocyte subsets. 

### 2.3. Rabbit

The rabbit is phylogenetically closer to primates than are rodents. Moreover, the rabbit provides an animal model that resembles humans in that the lung is the target organ for anaphylactic response. This species demonstrates both early and late phase airway responses, thus allowing mechanistic investigation of each reaction and the relationship between them. The latter's association with inflammation is thought to be of great importance in the development of asthma [9]. In order for rabbits to develop late phase airway response, neonatal immunization is required [7].

From an immunological point of view, rabbits produce IgE as the primary anaphylactic antibody. The presence of IgE is necessary to initiate antigen-induced late responses in the rabbit's lungs [24]. Rabbits are easy to handle and readily available, making them a good model for investigating lung-related disease. In addition, rabbits are large enough to allow nonlethal monitoring of physiological changes. 

Despite the many advantages of the rabbit as an animal model, this species has not been widely used, probably due to limitations in terms of cost and reagent availability. The cost of the animal itself as well as the space required to house it makes the rabbits more expensive to use than smaller species such as guinea pigs or mice [7]. In addition, gene sequences for rabbits are not well mapped at this time. 

Due to the low numbers of knock out or transgenic species and the lack of rabbit-specific reagents, mechanistic rabbit studies are also limited, particularly those involving genetics [7]. A few transgenic rabbits are available, but most studies involving the use of transgenic rabbits have focussed on pulmonary, cardiovascular, and metabolic issues [25].

This paper focuses on the use of rabbits in modeling both acute and chronic lung diseases and as a model for stem cell therapy. When considering the use of rabbits, there are several distinct components that need to be considered, especially anatomical structure of the rabbit airway and the ability of the rabbit to imitate human lung disease as much as possible.

## 3. Background on the Use of the Rabbit as a Disease Model

Mimicking the human condition in the animal model is decisive and critical to obtain accurate and comparable results [26]. Thus, it is challenging to choose the right animal model system. One of the most crucial aspects to consider when choosing the suitable model is anatomical structure. The lung is a sac organ that functions to contain and allow exchange of respiratory gases. It is a complex structure consisting of tracheobronchial branches and cellular parenchyma, which drive the dynamic physiological mechanism of the lung. Thus, these anatomical features of the lung must be compared between humans and rabbits to evaluate the ability of the rabbit animal model to recreate human chronic lung disease (Table 3).

### 3.1. Respiratory Bronchioles

Respiratory bronchioles (RBs) are important airway anatomical structures that differ between species. RBs are partially alveolarised airways located between the terminal bronchioles and the alveolar ducts. Absence of RBs enable the clearance of insoluble particles from alveolar ducts directly to terminal bronchioles. These were proved to be efficient. Clearance failure results in the pathological condition. Unlike rabbits, humans possess RBs, which evidently play a role in the development of emphysema and fibrosis [27].

### 3.2. Tracheobronchial Capillary Bed

The airway mucosa is a relatively highly vascularised structure. In a pathological condition like asthma, vascular permeability changes contribute to mucosal thickness. At this point, inflammatory cells, chemical mediators, and plasma protein migrate from blood vessel to the interstitial tissue. Accumulation of these inflammatory substances leads to the oedema and mucosal thickness. The arrangement of the vascular circulation consists of the submucosal plexus interconnected with an outer adventitial plexus of vessels, which is located outside the smooth muscle and bronchial cartilage [28]. Rabbits have five capillaries per mm, whereas humans have seven capillaries per mm [29]. Compared to other species, humans and rabbits have a minimal capillary network.

### 3.3. Branching

The lower respiratory airway begins with bifurcation of the trachea into two bronchi, one leading to each lung. Both main bronchi further subdivide into their respective lung subsmenental lobar. Further branching into bronchioles occurs, with airway trees ending in terminal bronchioles. Terminal bronchioles are connected to alveolar ducts, where gas exchange took place [30]. Humans and rabbits have 25 and 32 generations of airway branching, respectively, [13, 31], and they exhibit a similar symmetrical branching pattern [14]. Each of the main branches divides into two asymmetrical daughter branches. Two daughter branches that have an identical diameter are referred to as dichotomous, whereas an imbalance of the diameter is referred to as monopodial. Branching in humans is almost perfectly dichotomous, whereas in rabbit the diameter of the airway branches is monopodial. The branching pattern may influence the distribution and deposition of air and particles [15].

### 3.4. Lung Volume

Changes in lung volume are related to lung dysfunction and disease impacts on lung mechanics, gas exchange, respiratory muscle function, the sensation of dyspnoea, and tolerance to maximum exercise. Monitoring of lung volume is crucial when conducting an experiment to study a pathological condition. In rabbits and humans, lung expansion exhibits similar growth from birth to adulthood, with a 20-fold and 26-fold increase, respectively [16, 17].

### 3.5. Airway Epithelial Layer

The airway is a pipe-like structure that is lined by a pseudostratified epithelial cell layer, and each cell plays a critical role in maintaining the passage of air. As a group, these cells act as a protective barrier and defence mechanisms against foreign air particulates [32]. Goblet cells, ciliated cells, and basal cells line the epithelial layer of the large proximal airways. In the more distal airways, goblet cells gradually are replaced by Clara cells. The composition of the respiratory epithelial cells varies among species. Cell density is one of the prominent differences between humans and rabbits. 

### 3.6. Mucus Producing Cells

In the airways, mucus is produced by goblet cells and submucosal glands. It is a main component of the mucus gel layer which is located at the apical epithelium. Mucus consists of water, electrolytes, antimicrobial substances, anti-inflammatory cells, and many other compounds. Thus, mucus serves as a neutraliser of foreign materials. Mucus overproduction is a main hallmark of pathological conditions, as it leads to obstruction of the airways. This is due to increased number of goblet cells which often called hyperplasia and mucus production rate. In humans, submucosal glands are distributed along the airway and are concentrated between the cartilaginous rings [19], thus making goblet cells are abundantly seen in the epithelial layer [19]. In contrast, rabbits do not have submucosal glands and goblet cells appear to be less abundant compared to humans [19]. Despite the absence of submucosal glands, the rabbit has been used in studies specifically focussed on changes in goblet cells that occur in response to a pathological condition [33]. 

### 3.7. Use of the Rabbit as a Model for Lung Disease

The rabbit is phylogenetically closer to humans than are rodents. Because of the anatomical, physiological, genetic, and biochemical similarities between rabbits and humans, this species is preferentially used in pulmonary, cardiovascular, and metabolic studies, including those of airway obstructive disease, embolic stroke, arteriosclerosis, cholera, and cystic fibrosis. As a classical experimental animal model, rabbits also are used for drug screening tests, antibody production, and the production of therapeutic proteins. 

## 4. Remodelling of Lung Disease in Rabbit

There are several distinct components that need to be considered when modelling lung disease in animal models. These include protocols for sensitisation and methods for measuring the extent of disease, such as lung inflammation, AHR, and others. 

### 4.1. Sensitisation Method

A number of allergens have been used in animal models of lung disease, especially asthma. In the 1970s, Pinckard et al. [18] introduced the method of intraperitoneal (i.p.) administration of antigen in combination with adjuvant to neonatal rabbits. They injected soluble bovine serum albumin in conjunction with *Corynebacterium parvum* adjuvant to newborn rabbits within the first 24 h of life, and this continued every week according to their study design. The regime resulted in the production of antigen-specific IgE antibodies. 

This protocol has subsequently been modified, and various antigens have been used to sensitise animals, especially rabbits. Among them are ovalbumin (OVA), lipopolysaccharide, house dust mite (HDM), and ragweed pollen [7]. Shampain et al. [24] conducted a study in which they sensitised rabbit neonates with *Alternaria tenuis* as the allergen. This regime leads to the development of early and late airway responses to acute antigen challenge. Douglas et al. [34] performed a study to determine the effect of long-term exposure of sensitised rabbits to environmental pollutant gases in conjunction with the aetiology of asthma. They sensitised different rabbits with two types of allergen: *A. tenuis* and HDM. In another study, researchers used a combination of allergens in a single animal model; animals were sensitised either with single, double, or triple allergens (HDM, ragweed, *Aspergillus fumigatus*) [34]. 

OVA has been widely used to sensitise and challenge host animals. As OVA is readily available and can be easily manipulated via diet such that animal's immune system has not been exposed to OVA before sensitization [22]. The OVA model of airway inflammation is usually characterised by high levels of OVA-specific IgE and eosinophils, a T-cell predominant bronchial inflammatory response, and the development of AHR [20], which are similar to characteristics of asthma in humans.

A typical sensitisation protocol usually involves injecting the allergen with adjuvants into the animal. Adjuvants such as aluminium hydroxide (alum) are responsible for inducing strong and sustained sensitisation, promoting the development of a Th2 phenotype by the immune system, and promoting production of allergen-specific IgE and IgG1 [22, 35]. Other adjuvants that have been used in sensitising animal models include heat-killed *Bordetella pertussis*, raisins, and adjuvant mixes such as Freund's complete adjuvant [22]. However, this adjuvant mix is known to promote a more Th1-biased response, which is not comparable with characteristics of human asthma. 

Conrad et al. [36] compared experimental asthma phenotypes between adjuvant and adjuvant-free protocols in inducing allergic airway inflammation in murine. The sensitisation protocol involved injection of antigens using two different routes (i.p. and subcutaneous (s.c.)). They found that the s.c. adjuvant-free protocol resulted in significantly higher OVA-specific IgE and significantly lower OVA-specific IgG1 levels than the i.p. adjuvant protocol. Differences in the levels of IgE and IgG1 between these two protocols resulted from the adjuvant itself. Alum participates in the generation of humoral immunity [36], thus it promotes high production of OVA-specific IgG1 antibodies in comparison to an adjuvant-free protocol. Conrad et al. [36] concluded that the s.c. adjuvant-free protocol generated a phenotype that was similar to the standard OVA i.p. adjuvant protocol used in the majority of studies. However, different adjuvants and their use or omission provide options for researchers when designing sensitisation protocols. 

### 4.2. Lung Function

To investigate physiological parameters such as lung function, it is very important to work with individual animals that can undergo repeated assessment during the time course of the study. Thus, it is important to use an animal for which longitudinal responsiveness can be investigated, as this would occur clinically in the investigation of human subjects with asthma. For this reason, the rabbit is a better model for investigating lung function compared to other animals. Rabbits can act their own control when measuring lung function, as repeated measurements can be made in rabbits using an endotracheal tube and oesophageal balloons. This technique is clearly impossible in smaller animals such as rats and guinea pigs, for which invasive surgery is needed to perform the investigation. In addition, anaesthetised rabbits remain breathing throughout the course of the experiment, thereby excluding the need for mechanical ventilation, which is required for smaller animals [7]. Thus, the rabbit model offers advantages for lung function studies.

### 4.3. Airway Hyperresponsiveness

AHR can be defined as an increase in sensitivity and reactivity of the airways in response to physical and chemical stimuli [37]. This AHR is considered to be the hallmark of asthma, and greater AHR has been correlated with increased disease severity [38]. Sensitised rabbit neonates have shown an enhanced responsiveness to various stimuli, including histamine, methacholine, and adenosine 5′ monophosphate (AMP) [7]. AMP causes a marked dose-related bronchoconstriction that may occur within minutes to an hour if inhaled by atopic asthma patients. AHR to inhaled AMP may reflect the severity of airway inflammation, and this phenomenon has been demonstrated in various animal species. In 1996, El-Hashim et al. [39] reported bronchoconstriction in response to inhaled adenosine in allergic rabbits, and it appeared to be mediated by the activation of A_1_ receptors. In another study using the allergic rabbit model, Obiefuna et al. [40] described the role of the A_1_ adenosine receptor antagonist L-97-1 in reducing antigen-induced early and late responses and the allergen-induced hyperresponsiveness to inhaled histamine and adenosine.

### 4.4. Early and Late Phase Airway Responses

Rabbits can develop both early and late phases of airway response, but neonatal immunisation is required for the late phase response to be developed. Herd et al. [41] reported that neonatal rabbits immunised within 24 h of birth [41] exhibited many features of human asthma, including AHR in response to inhaled antigen, acute and late phase airway obstruction [24], pulmonary eosinophil and lymphocyte recruitment [41, 42], and production of IgE antibodies [24, 43]. This clearly shows the ability of the rabbit to develop disease from the point of being sensitised through adulthood, thus making this species ideal as a model for human asthma.

Theoretically, bronchial allergen challenge in sensitised atopic asthmatics will lead to both acute and late phase airway obstruction. The acute phase occurs as a result of contraction of the smooth muscle of the airway, whereas the late phase occurs due to inflammation. Studies have shown that adult rabbits that have been neonatally immunised to an antigen undergo both early phase and late phase airway obstruction in response to acute exposure of the airways to an aerosolised antigen [24, 44]. This finding is far different from what is seen in rats. Even though rats demonstrate both acute and late phase airway constriction, the severity of bronchoconstriction depends on the antigen used and is correlated with the titre of specific IgE antibody in the serum [45]. Bronchoconstriction is rarely provoked if the IgE titre, assessed using the passive cutaneous anaphylaxis assay, is lower than 16 [9].

In addition to the early response, allergen challenge of atopic subjects with asthma leads to AHR 24 h later [7]. The late response usually is associated with an increased responsiveness of the airways to various stimuli, including inhaled histamines and methacholine. Neonatally immunised adult rabbits exhibit similar hyperresponsiveness after 24–48 h following allergen challenge. In contrast, mice do not demonstrate spontaneous AHR, as they fail to develop an airway constrictive response to histamine [8]. In particular, it is unclear whether mice are capable of exhibiting a physiological late phase constriction in the lung [46, 47].

### 4.5. Airway Inflammation

Inflammation plays a key role in human asthma. Asthmatic patients usually exhibit signs of inflammation, such as infiltration of inflammatory cells into lung tissues, high levels of IgE and IgG1 in sera, thickening of airway epithelial tissue, and excessive mucus secretion due to goblet cell hyperplasia and hypertrophy of mucous glands [48]. In the case of allergic rabbits, following allergen challenge, bronchoalveolar lavage (BAL) may be performed to assess the extent of cell infiltration. Typically lung lavage is performed using saline via an endotracheal tube. The collected BAL fluid is used to determine both total and differential cell counts [7]. In addition, the pathology of asthma in allergic rabbits can be observed histologically.

The most important pathological change that occurs in the lung during injury is the infiltration of neutrophils into the lung. During the hypersensitivity response, inflammatory cells are recruited into affected sites. Neutrophils are the first cells to be recruited to the site of inflammation, and they have potent antimicrobial armour, which includes oxidants, proteinases, and cationic peptides [49]. Activated neutrophils and alveolar macrophages secrete inflammatory mediators that disrupt epithelial fluid transport and impair surfactant production by alveolar type II cells [10]. Several studies of this process have been conducted using animal models, such as neutrophil assessment during ALI/ARDS onset in rabbits [50]. In a study using the rabbit as an *in vivo* model, Folkesson et al. [51] showed that neutrophil recruitment into the area of inflammation was highly regulated by the presence of interleukin-8 (IL8). 

## 5. The Potential Use of the Rabbit Model in Drug Screening Tests

Animals have been widely used for drug screening tests (e.g., in order to predict the safety and effectiveness of a drug before it is used clinically). The allergic rabbit model has been extensively used in assessing various antiasthmatic drugs. In general, the allergic rabbit is sensitive to similar drugs as human patients with asthma. Clinically, isoprenaline has been reported as not being able to reverse the late reaction in asthmatic patients once it developed [24]. In the preclinical setting, this drug also is unable to induce any effect on the increase in airway resistance or the decrease in dynamic lung compliance in the allergic rabbit model [7].

 Administration of sodium cromoglycate in the allergic rabbit prior to allergen challenge was shown to inhibit both early and late phase responses. This finding has also been clinically shown in asthmatic patients, as discussed by Altounyan [52]. Administration of xanthines in humans inhibits the development of the late response following allergen challenge. In rabbits, aerosolised theophylline was shown to inhibit both the early and late phase of bronchoconstriction induced by antigen as well as AHR [7]. 

Peptide leukotrienes can induce many features of asthma in both humans and experimental models, including airway obstruction, mucus secretion, increased vascular permeability, inflammatory cell infiltration, and AHR [7]. The effect of PF 5901, a leukotriene synthesis inhibitor and LTD_4_ antagonist, had been tested in rabbits, and it has been reported that the drug had no effect on acute bronchoconstriction or eosinophil infiltration. Nevertheless, AHR was inhibited and levels of LTD_4_ were reduced following the antigen challenge [41]. 

Corticosteroids can be used to treat ALI/ARDS in asthma patients. The administration of the corticosteroid budesonide in sensitised rabbits resulted in marked anti-inflammatory activity and inhibited antigen-induced AHR and bronchodilator responses [53]. Budesonide also can inhibit the early and late responses as well as eosinophil infiltration [7].

## 6. The Rabbit as a Model for Cell Therapy Research

### 6.1. Airway Epithelium Injury

Various methods have been developed to target the damage to particular regions of the airway due to inhalation or iatrogenic causes [54]. Inhalation of foreign substances into the lungs can block the airway and also interfere with gas exchange directly by physical obstruction or indirectly by provoking acute bronchospasm or delayed inflammation. Iatrogenic injury due to intubation can cause upper airway obstruction from oedema. Both injuries caused disruption of cells that can be classified into four categories: (i) reversible injury to the airway which will heal and return to normal condition once the damage is removed; (ii) exfoliation of individual cells with the majority of nonciliated columnar and basal cells left intact; (iii) desquamation of groups of cells but with the basal cell layer left intact; and (iv) desquamation of cells, including the basal cells [55]. 

### 6.2. Repair Process following Injury

Under normal circumstances, the damaged epithelium is able to repair itself rapidly. Immediately after injury occurs, the airway epithelium initiates a repair process in order to restore the integrity of the barrier [56]. The cells that participate in wound healing and functional regeneration of the epithelium in both humans and rabbits are the epithelial basal cells of the trachea and bronchi, Clara cells of the bronchioles, and alveolar type II (ATII) cells [57]. 

The regeneration process is a complex phenomenon that quickly starts after the lesion occurs. These cells can rapidly change their structure and function in order to adapt to changes in the local environment or to repair the epithelium after injury. *In vitro *studies of human airway epithelium cell restitution have shown that the regenerative process to repair the airway epithelium involves dedifferentiation, migration of neighboring epithelial cells to cover the denuded area, proliferation of progenitor cells to restore cell numbers, and redifferentiation to restore the function of epithelial cells [55, 57–59]. 

### 6.3. Stem Cells and Progenitor Cells

Stem cells are defined as unspecialized cells that have the remarkable potential to develop into varying types of cells. As an internal repair system, these cells can replenish injured cells. For cells to fall under the definition of stem cells, they must have the ability of unlimited self-renewal to produce progeny that is exactly the same as the originating cell, and they must be able to give rise to a specialized cell type that becomes part of the organ targeted [60]. Most organs contain their own small reservoir of stem cells, also called progenitor cells, which are recruited to start dividing and replace cells that have died during normal aging or to repair small areas of damage [61]. Stem cells also can differentiate into progenitor cells, which are lineage-specific precursors to a more restricted development potential [62]. Several potential sources of progenitor cells for airway epithelium have been identified and are divided into two groups: endogenous stem cells and exogenous stem cells [63].

### 6.4. Endogenous Stem Cell

Endogenous stem cells are already present in the respiratory tract. In both rabbits and humans, the basal cells are the primary stem or progenitor cells in the airway, and the ability to differentiate into other types of cells such as secretory or ciliated cells can be detected by using cell proliferation markers [64]. Clara cells which are located in the bronchioles are believed to be capable of returning to proliferation after injury [65]. In the gas-exchanging region of the lung, ATII cells are the progenitor cells and can differentiate into ATI-like cells [66]. Collectively, these studies have shown that stem or progenitor cells are recruited into the injured area. The cell proliferation and phenotypic differentiation also lead to recovery of epithelium function.

### 6.5. Exogenous Stem Cell

The repair ability possessed by endogenous lung epithelial progenitor cells is often insufficient as the natural repair capacity appears to diminish with age [67]. Direct interface with the outside environment makes epithelial cells inside the lungs susceptible to potential toxic agents and pathogens, thus they must be able to respond quickly and effectively to cellular damage [68]. Although the damage can be rapidly and completely repaired by progenitor cells, these cells are usually present in insufficient numbers to compensate for extensive damage [61]. The purpose of stem cell transplantation is to replace damaged or lost cells in an organ or tissue. Autologous or allogeneic cell transplantation in experimental models has shown that these cells can engraft in the lung and differentiate into mature epithelial phenotypes [69, 70] and thus increase the cellular response to injury [71].

### 6.6. Cell-Based Therapy

Cell-based therapy has attracted tremendous interest recently. The concept of regenerative medicine using cells to repair tissues represents the potential to develop alternative therapeutic strategies that may ultimately play a major role in the treatment of a number of diseases [72]. There are many possibilities for cell-based therapy depending on the disease itself. Given the ability of regeneration, cell-based delivery offers new potential for treating diseases such as COPD and asthma. Much still is needed to be learned about their characteristics, manipulation, safety and application of these cells for effective cell-based therapy to treat diseases. Moreover, for cells to be successfully used for human treatment, precise delivery and targeting of the cells to the site of the disease must be ensured.

An overview of *in vivo* studies of cell-based therapy that used the rabbit as an animal model is provided below. Most of these studies indicated that this kind of treatment is feasible, safe, and likely to be applicable in clinical trials in the near future.

### 6.7. Bone-Marrow-Derived Stem Cells

Adult stem cells, such as hematopoietic and mesenchymal stem cells (MSCs), are found in mature bone marrow tissues. Plasticity of adult stem cells means that they can generate lineages of cells that are different from their origin. Thus, these cells can be used for organ regeneration and for cellular repair in various animal species as well as in humans [72]. The ability to differentiate into various mesodermal cells makes MSCs the most commonly used adult stem cells in cell-based therapy. MSCs can differentiate not only into mesenchymal lineage cells but also into endothelium and endoderm *in vitro* [73]. However, MSCs also can be organ specific, as populations isolated from various sources might be functionally different although morphologically similar. For example, MSCs isolated from the umbilical cord do not have the same ability to give rise to osteoblasts, chondrocytes, and cardiomyocytes as bone marrow-derived MSCs [74]. Three basic mechanisms have been proposed to explain how MSCs can repair tissue injury: (a) creation of a milieu that enhances regeneration of endogenous cells; (b) transdifferentiation; or (c) cell fusion [75–77]. Because of their potential use in regenerative medicine, the therapeutic potential of MSCs has been explored through autologous or allogeneic transplantation in patients through local delivery or systemic infusion [78]. 

Zhu et al. [79] studied the effect of MSC engraftment on vascular endothelial cell growth factor (VEGF) in rabbit lung tissue, plasma, and extravascular lung water at an early stage of smoke inhalation injury. MSCs inhibit the activation and proliferation of immune cells by secreting an inhibitory factor to reduce the production of factors that induce the production of VEGF. VEGF itself participates in an inflammatory reaction, formation of pulmonary oedema, and aggregation of neutrophilic granulocytes in the lung. Thus, the engraftment of MSCs had a protective effect on smoke inhalation injury through regulation of the local and systematic VEGF and reduction of lung water content.

Haematopoietic stem cells are progenitors for several cell types, such as endothelial cells, epithelial cells, myocytes, and neurons, and they also are able to reduce lung fibrosis [80]. Bone marrow mononuclear cells (BMnCs), which are known to contain both haematopoietic cells and MSCs, were shown to have therapeutic potential for acute lung injury [81] and lung fibrosis [82, 83]. BMnCs may improve lung injury due to bone marrow cell differentiation into epithelial and endothelial cells [81]. Yuhgetsu et al. [84] used autologous BMnCs to mitigate elastase-induced pulmonary emphysema in rabbits. The cells were transplanted via the left and right main bronchi; 4 weeks after administration, the rabbits showed significantly better pulmonary function and smaller alveolar airspaces. This inhibition of progression of emphysema was due to the BMnCs' ability to attenuate inflammation, MMP-2 expression, and apoptosis while enhancing alveolar cell proliferation.

### 6.8. Endothelial Progenitor Cells

Endothelial progenitor cells (EPCs) can be isolated from peripheral blood or bone marrow. They react to physical and chemical stimuli within the circulation and regulate homeostasis, vasomotor tone, and immune and inflammatory responses [85]. Circulating EPCs play important roles in angiogenesis [86] and repair of injured endothelium [87]. Circulating EPCs are mobilized from the bone marrow to peripheral circulation by cytokines, growth factors, and ischemic conditions during endothelial injury [86].

Lam et al. [88] studied circulating EPCs as a potential therapeutic treatment for ALI. One week after culturing EPCs, ALI was induced in rabbits by oleic acid, and autologous EPCs were then transplanted intravenously. The EPCs preserved pulmonary endothelial function and maintained the integrity of the pulmonary alveolar-capillary barrier. The paracrine effect of EPCs due to secretion of growth factors contributed to the overall beneficial effect on endothelial repair. Another study using circulated EPCs from peripheral blood mononuclear cells was designed using the rabbit as a model. He et al. [89] demonstrated that after 4 weeks, transplantation of autologous EPCs enhanced endothelialisation and improved endothelial function of the denuded carotid artery.

### 6.9. Tissue Engineering

Tissue engineering involves creating whole organs or parts of organs to replace and repair damaged tissues. Constructing three-dimensional tissues from two-dimensional cell layers outside the body is a major challenge that requires very specific knowledge and skills. The process involves three important steps. First, the desired cells, which can be derived from stem cells, must be obtained. However, the stem cells must differentiate into the targeted cells first. Second, the cells need a structure or scaffolding on which to attach, grow, and carry out their specialized function (e.g., producing bone or cartilage proteins). Last, the three-dimensional tissue-engineered organ part should have an adequate supply of oxygen and cell nutrients [61].

Tissue engineering presents a promising technique to create a functional organ substitute. In one study, a tissue-engineered trachea fabricated from fibrin gel and autologous chondrocytes was transplanted into rabbits; successful regeneration and functional restoration of ciliated epithelial cells on the operated site were achieved without graft rejection and inflammation [90]. Rabbits also have been used to study airway stem cell biology and lineages using purified or enriched specific epithelial cell types to reconstitute denuded tracheal grafts. Inayama et al. [91] demonstrated the ability of rabbit basal cells to repopulate epithelium containing basal, ciliated, and goblet cells after reconstitution of a tracheal xenograft.

## 7. Conclusion

Overall, the rabbit is a useful model for studying lung physiology and pathophysiology. The rabbit as a model offers better understanding of lung structure than smaller animal models. The rabbit model permits investigation of chronic aspects of disease because it can act as a control by allowing multiple measurements to be made at different time points. The ability of the rabbit to develop diseases once sensitised with several stimuli at birth provides opportunities to investigate the risk factors that contribute to allergic disease. The most important aspect of the rabbit model for lung disease is its similarities to humans in terms of asthma development; thus, it provides an ideal basis studying the effects of novel asthma therapies [7].

Remarkable progress has been achieved in the study of stem cells as avenues to provide clinical deliverables. Stem cell therapy represents a fascinating new approach for the management and repair of injuries. Recent *in vivo* studies using rabbit models have shown the feasibility of autologous and allogeneic cell therapy. However, current studies are in the early stages, and there is still much to be learned, such as how to enhance production, survival, and integration of transplanted cells prior to realistically contemplate clinical strategies.

Although the rabbit as a model has several limitations, these limitations can be overcome with modification and improvement. It is important to remember that a rabbit or any other animal model cannot be considered as a surrogate for human disease. Instead, such models should be seen as an important opportunity to generate and test hypotheses in a simple and controlled system. The clinical relevance of the findings in rabbits and other species can only be determined in human studies [8].

## Figures and Tables

**Table 1:** Characteristics of human lung injury.

Clinical features	Acute onset
	Diffuse bilateral alveolar injury
	Acute exudative phase
	Repair with fibrosis

Physiological changes	V/Q abnormalities
	Severe hypoxemia
	Decreased compliance
	Impaired alveolar fluid clearance

Biological changes	Increased endothelial and epithelial permeability
	Increased cytokine concentration in lungs
	Protease activation
	Coagulation abnormalities

Pathological changes	Neutrophilic alveolar infiltrates
	Intra-alveolar coagulation and fibrin deposition
	Injury of the alveolar epithelium with denudation of the basement membrane

**Table 2:** Advantages and disadvantages of various animal models for studying lung disease, particularly asthma [8, 9].

Animal	Advantages	Disadvantages
Mouse	IgE is the major anaphylactic antibody	Do not exhibit spontaneous AHR
	Numerous inbred strains	Limited airway musculature
	Numerous immunological reagents	Lung anatomy differences
	Small, relatively inexpensive	Do not easily demonstrate smooth muscle hyperplasia

Guinea pig	The lung is the primary organ of anaphylaxis	IgG1 is the major anaphylactic antibody
	Show early and late phase airway responses	Shortage of inbred strains
	Small, docile animals, inexpensive	Few species-specific reagents

Rabbit	Phylogenetically similar to humans The lung is the major target organ IgE is the major anaphylactic antibody Large enough to study for lung mechanics Demonstrate both early and late phase airway responses	Neonatal immunisation required for developing late phase airway response Few species-specific reagents Few transgenic species available Gene sequences are not well mapped

**Table 3:** Anatomical comparison between the human and rabbit lung.

Anatomical structure	Human	Rabbit	Reference(s)
Respiratory bronchioles	Present	Absent	[13]
Tracheobronchial capillary bed	7 capillaries/mm	5 capillaries/mm	[14, 15]
Branching pattern	More symmetrical 25 generations	Less symmetrical 32 generations	[16–18]
Lung expansion rate	26 folds	20 folds	[16, 17]
Mucus producing cells	Submucosal glands and goblet cells	Only goblet cells	[19]
